# Preferred mode of delivery association with the body image and genital image in pregnant women - a cross-sectional study

**DOI:** 10.1186/s12884-023-05589-3

**Published:** 2023-07-04

**Authors:** Hamideh Khosravi, Zahra Mehrbakhsh, Sedigheh Moghasemi, Ghazale Samiei

**Affiliations:** 1 Counseling and Reproductive Health Research Centre, Department of Midwifery, School of Nursing and Midwifery, Golestan University of Medical Sciences, Gorgan, Iran; 2 Department of Biostatistics , School of Public health, Hamadan University of Medical sciences, Hamadan, Iran; 3 Department of Biostatistics and Epidemiology, School of Public health, Golestan University of Medical sciences, Gorgan, Iran

**Keywords:** Delivery, Obstetric, Body image, Natural childbirth, Pregnancy, Genital image

## Abstract

**Background:**

Pregnant women experience several changes in their appearance, body shape and body image. In some studies, there has been a relationship between these changes and the type of delivery. This study aimed to investigate the relationship of the prenatal body image and genital image with the mode of delivery preferred by pregnant women in Gorgan in 2020.

**Methods:**

In this cross-sectional study, 334 pregnant women were selected by stratified sampling. The Prenatal Body Image Questionnaire (PBIQ), Female Genital Self-Image Scale (FGSIS), pregnant women’s preferences for mode of delivery questionnaire (PPMDQ) and DASS-21 were completed on line. The data was analyzed using Spearman test and linear regression.

**Results:**

The average score of PBIQ, FGSIS, and PPMDQ was 68.24 (standard deviation = 17.71), 19.25 (standard deviation = 3.3), and 63.12 (standard deviation = 3.3) respectively. Vaginal delivery as a preferred mode of delivery was inversely correlated with dissatisfaction with body image (r=-0.32, P < 0.001), and directly correlated with satisfaction with the genital image (r = 0.19, P < 0.001). There was a significant inverse correlation between prenatal body image dissatisfaction and genital image satisfaction (r=-0.32, P < 0.001). While FGSIS score could not predict PPMDQ, PBIQ score could.

**Conclusions:**

Satisfaction with the prenatal body image or genital image is associated with the choice of vaginal delivery. These results can be the basis for prenatal care and childbirth counselling.

**Supplementary Information:**

The online version contains supplementary material available at 10.1186/s12884-023-05589-3.

## Background

Body image refers to anyone’s perception of the size and shape of their body and also the feeling and attitude they have towards each part of it [1]. Pregnancy as an unique experience for women is associated with rapid and significant changes in the body size and shape that push women further from ideal body shape which can be assigned as desirable or undesirable [2, 3]. Specific changes in pregnancy to the body that cause dissatisfaction, including; an increase in breast, abdomen, hip, legs and thighs size, and undesirable skin and hair changes such as acne, stretch marks, cellulite, varicose veins, thicker or oily hair. While some report desirable body changes including developing the pregnancy ‘glow’ and skin improving, thighs appearing smaller in size, and an increase in breast size for those women who characterized themselves as small breasted pre-pregnancy [3]. Moreover, If changes considered natural, related to pregnancy and as a sign of fetus normal growth, and along with the positive feedback of the husband some women are satisfied with their body changes [3, 4]. Therefore, changes in body image are prevalent in pregnancy [5, 6] and satisfaction with it can be diverse [7] according to the biopsychosocial factors.

Dissatisfaction with body image consequently could result in, some psychological health problems such as anxiety, depression, social isolation and weakening of self-concept and self-esteem [8]. It might also affect the health and quality of life of pregnant women [9]. While, body image satisfaction in pregnan women is associated with general and sexual self-esteem [10] and sexual function [11, 12].

Dissatisfaction with body image may also include the genital area. According to Gomes et al., the highest level of women’s dissatisfaction with their body was associated with dissatisfaction with genital image [13]. While some researchers believe that genital image measures an aspect of the body image that is different from the general body image [14]. Olsson et al. (2005) reported that after a vaginal delivery women considered their vagina larger and looser than previous, while their husbands did not [15]. Dissimilarity of women and their husbands’ views might indicate how the actual shape and function of the female genitalia is different from the genital self-image. In line with this study, others addressed that maintaining the beauty and functionality of the genitalia by preventing loosening and damage to the perineal muscles [11, 16] and, thus, preventing sexual dysfunction [1, 11, 16] is one of the most important reasons for women to choose cesarean section [11]. In this regard, the prevalence of fear of injury and rupture of the genitals during labor has been reported to be more than 50% [17].

According to the available studies, body image seems to be one of the factors affecting the preferred mode of delivery in women, and there are differences in the important dimensions of the body image in the two groups of pregnant women selecting cesarean section and vaginal delivery. In a study, pregnant women who had selected cesarean section were more concerned about bladder and uterine prolapse, while in women choosing vaginal delivery, the general dimensions of the body image, such as acceleration of the body’s return to its previous state and leaving no cesarean scars on the body were more considered [18].

While some studies have referred to the correlation between body image and genital image in women [13, 19], no significant relationship has been found between these two in other studies [14]. A study conducted in Indiana University found that women with a history of vaginal delivery were more satisfied with their body image and less satisfied with their genital image than women who had a cesarean section. However, it is not clear whether this difference existed also during pregnancy or not [14].

The rate of maternal and neonatal mortality in cesarean section is several times higher than vaginal delivery [20, 21] and the latter is supposed to be the best mode of delivery in most pregnancies. Nonetheless, nowadays, the demand for cesarean section has increased for various reasons such as anxiety and worry about genital rupture, changes in sexual relations, and insistence of spouse [20, 22]. Therefore, women’s body image disorder during pregnancy or after childbirth seems to be one of the important factors affecting their preferred mode of delivery.

Considering the increasing rate of caesarian section in the world [23] there is a call for investigation of non-medical reasons including social, cultural and psychological factors [24]. Few studies have been conducted on the relationship of the body image and genital image satisfaction with the preferred mode of delivery in pregnant women [25]. Additionally, although body image is affected by socio-cultural factors, most related studies have been conducted in English-speaking countries [3], indicating the need for such studies in other countries. Accordingly, the aim of this study was to determine the relationship of the body image and genital image with the preferred mode of delivery in pregnant women in Gorgan, Iran.

## Methods

### Study design and setting

In this descriptive cross-sectional study, 334 married pregnant women (gestational age of 30–37 weeks) were selected by stratified sampling method with proportional allocation from Gorgan (The center of the Golestan province, in the north of Iran) Comprehensive Urban Health Service Centers. All participants were Iranian and Muslim, and as they have selected from governmental health services, they mostly belonged to the middle to low socioeconomic class. Sampling was performed from May 2020 to October 2020. It should be noted that the present study reported based on Strengthening the Reporting of Observational studies (STROBE) [26].

### Sample size determination and participants

In this study, the maximum sample size was determined by considering the assumption that there is a linear relationship between body image and genital image, the correlation coefficient in the null hypothesis (-0.4) with 95% confidence level and test power of 80%, a 10% drop in samples as well as according to the inclusion criteria and using the results of the meta-analysis conducted by Azami et al., which reported a 15% prevalence of moderate to severe depression in Iranian pregnant women [27]. Thus, the sample size was determined based on the number of pregnant women referred to Gorgan comprehensive Health Centers, using stratified sampling method with appropriate allocation (Additional file 1: Appendix table).

**Fig. 1:**
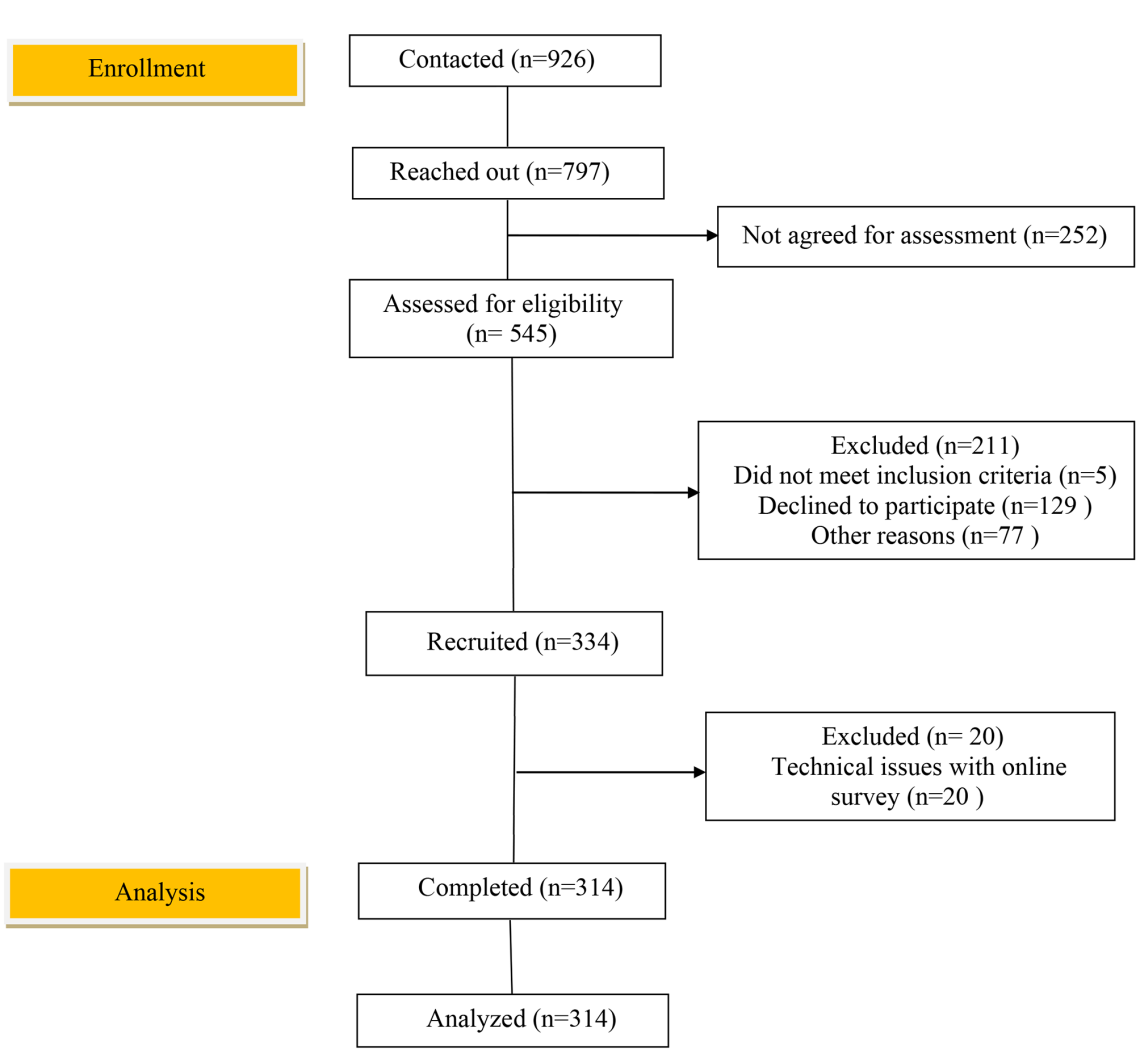
Flow chart depicting the process of sampling in the study of Relationship of the body image and genital image with the preferred mode of delivery in pregnant women: a cross-sectional study

To this end, after obtaining the necessary permission for being in the research setting, the researcher went to the comprehensive urban health service centers of Gorgan city in order to collect data. In each center, all eligible mothers were invited to study based on the information available in the NAB health registry system. Inclusion criteria were singleton pregnancy, gestational age of 30–37 weeks [28], and education level of higher than fifth grade. Exclusion criteria included detected fetal abnormalities, mental or motor disabilities in the woman, diagnosed sexual dysfunction (self-report), and acheiving a score 11 and higher in depression subscale of DASS-21. Figure 1 depicting the process of sampling in the study.

## Materials

Data were collected using web-based electronic questionnaire (https://porsline.ir/) including demographic and reproductive form, and the Persian versions of Prenatal body image questionnaire (PBIQ), Female Genital Self-Image Scale (FGSIS), Pregnant women’s preferences for mode of delivery questionnaire (PPMDQ), and Depression Anxiety Stress Scale (DASS-21). The demographic and fertility form consisted of 40 questions (12 demographic questions and 28 clinical questions and questions related to the fertility history).

The Persian version of PBIQ was designed and psychometrically evaluated by Sohrabi et al. in 2019. This 30-item questionnaire includes seven sub-domains of fitness and beauty, lower body fat, attention to changes in pregnancy, shame, sexual attractiveness, negative feelings about skin changes and symbol of motherhood symptoms. Each item is answered based on a 5-point Likert scale (1 = strongly disagree to 5 = strongly agree), and higher score reflects more dissatisfaction with the BI in pregnancy. The achievable score range is between 30 and 150. Lower scores indicate less body-related concerns and more satisfaction with body image, while higher scores are indicative of greater dissatisfaction with body image [29]. In other word, the scale score could interpret from completely satisfied to completely dissatisfied. There is no cut-off point. In this study achievable score divided into quartile. Cronbach’s alpha of the questionnaire in Sohrabi study [29] and the present study was similar and equal to 0.92.

Female Genital Self-Image Scale (FGSIS) was designed and psychometrically evaluated by Herbenick and Reece in 2010 in order to assess the female genital self-image [30, 31]. The scale was translated to Persian and was psychometrically evaluated by Pakpour et al. in 2013 [19]. Similar to the original version, the P-FGSIS consists of seven items and two sub-domains of intrapersonal and interpersonal concerns. The items are scored based on a 4-point Likert scale ranging from 1 to 4. The achievable scores range from 7 to 28 and higher scores indicate a more positive image of the genitals. The scale has no cut-off point [19]. Cronbach’s alpha coefficient for the whole scale was 0.86 in the study of Pakpour et al. [19] and 0.6 in the present study. This scale was used in Jawed-Wessel et al. [14] and Komarnicky et al. [32] study.

The Persian version of pregnant women’s preferences for mode of delivery questionnaire (PPMDQ) has been designed and psychometrically evaluated for assessing the preferences of pregnant women for the mode of delivery. The questionnaire is a 21-item self-report scale in seven domains of self-efficacy, false impression of the C-section delivery benefits, exaggerating the risks of vaginal delivery, perceived susceptibility, normative beliefs, desire for acceptance and health professionals’ idea. The items are scored based on a 5-point Likert scale ranging from 1 to 5. The total achievable scores range from 21 to 105 and there is no cut-off point. For ease of interpretation, all questions have been scored in favor of vaginal delivery and a higher score shows the preference towards vaginal delivery [33]. Cronbach’s alpha coefficient for the whole scale was 0.74 in the study of Zmani-e-Alavijeh et al. [33] and 0.83 in the present study. This scale was applied in previous studies in Iran [34–36].

The short form of the Depression Anxiety Stress Scale (DASS) of Lovibond P.F and Lovibond S.H [37] includes 21 questions and three 7-items subscales of anxiety, depression and stress. The items are scored based on a 3-point Likert scale ranging from 1 to 3. The total achievable scores range from 0 to 21, and the higher the score, the worse is the situation. The Persian version of the short scale has been validated by Sahebi et al. Cronbach’s alpha coefficient for the depression scale was 0.77 in the study of Sahebi et al. [38], and 0.75 in the present study. Given the relationship between mental health of pregnant women and body image [6] and genital image [39], achieving a score lower than 11 for depression [38] was considered as one of the inclusion criteria showing the absence of depressive disorder. This scale was used for evaluating psychological/mental health in pregnant women [27, 39].

### Procedures and ethical consideration

This study was approved by the Research Ethics Committee of Golestan University of medical sciences.

### Data processing and analysis

The data were analyzed using descriptive statistical methods and comparison of means tests (for independent groups and quantitative variable), chi-square test (to compare qualitative variables) and regression in SPSS version 16. The value of p < 0.05 was considered significant in this study.

## Results

### Sample and demographic characteristics of participants

Out of 334 completed questionnaires, 17 questionnaires (5.09%) because of moderate and severe depression and 3 questionnaires (0.9%) for not answering to all questions were excluded from the study. Finally, the data of 314 subjects (94.01%) were analyzed.

The demographic and clinical characteristics of the samples are shown in Table 1. The mean age of the pregnant women was 29.3 years, the duration of their marriage was 6.63 years, and the mean years of their education was 13.57 years. Most women were housewives and their spouses had an average income. About half of the subjects were nulliparous (54.1%) and the rest were multiparous.

**Table 1:** Demographic and reproductive characteristics of pregnant women

Characteristic		Mean ± SD
Age (year)		**29.3± 5.3**
Duration of marriage (year)		**6.63 ± 4.62**
Education (year)		**13.57 ± 3.38**
Body mass index (BMI)**		**25.55 ± 4.87**
**Characteristic**		**n (%)**
Job	Housewife	**244 (78)**
	Employed	**69 (22.1)**
Family Income status *	Fair (> 37,000,000 Rials)	**41 (13.5)**
	Medium (18,500,000–37,000,000 Rials (	**137 (45.2)**
	Low (< 18,500,000 Rials)	**114 (37.6)**
	No income	**11 (3.6)**
Gravid	Primigravida	**143 (45.5)**
	Multigravida	**165 (55.5)**
Parity	Nulliparous	**170 (54.1)**
	Multiparous	**144 (46.9)**
History Abortion	No	**232 (73.9)**
	Yes	**82 (26.1)**
Type of previous childbirth	NVD	**78(24.8)**
	Cesarean section	**78(24.8)**
	Both	**4(1.3)**
	None	**154(49)**

* The minimum labor income in 2020 was equal to 18,500,000 Rials.

** Based on weight before or during first trimester of pregnancy

**SD: standard deviation**

**n: Number of participants**

### Correlations among the PBIQ, PPMDQ, and FGSIS

The mean and quartile frequency of the variables, including preferred delivery method, prenatal body image and genital image, are given in Table 2. The majority of pregnant women (89.5%) were satisfied with their body image. Most of them (76%) had a high satisfaction with their genital image; and the preferred mode of delivery in 48.5% of the subjects was probably or definitely vaginal.

**Table 2:** Mean and Frequency of quartile levels of main variables in pregnant women: Prenatal body image (PBIQ), Female Genital Self-Image (FGSIS) and Preferred mode of delivery (PPMDQ) in pregnant women

Characteristic	Mean ± SD	Group	Quartile	Frequency (%)
**PPMDQ**	63.12 ± 9.83	Definitely C/S	21 − 42	**9 (2.9)**
		Probably C/S	42.1–63	**152 (48.6)**
		Probably NVD	63.1–84	**146 (46.6)**
		Definitely NVD	84.1–105	**6 (1.9)**
**PBIQ**	68.24 ± 17.71	Completely Satisfied	30–60	**114 (36.3)**
		Satisfied	61–90	**167 (53.2)**
		Dissatisfied	91–120	**33 (10.5)**
		Completely dissatisfied	121–150	**0**
**FGSIS**	19.25 ± 3.3	Low satisfaction	7–12.25	**9(2.9)**
		Moderate satisfaction	12.26–17.50	**66 (21.2)**
		High satisfaction	17.51–22.75	**198 (63.5)**
		Very high satisfaction	22.76–28	**39 (12.5)**

The correlation between prenatal body image and the preferred mode of delivery in women was assessed using Spearman test the result of which is shown in Table 3. There was a significant inverse correlation between prenatal body image score and the preferred mode of delivery (p < 0.001, r = -0.325). In other words, more satisfaction with prenatal body image was associated with choosing vaginal delivery method.

As can be seen in the Table 3, since PBIQ measures prenatal body image dissatisfaction (it means Higher score means higher dissatisfaction), its correlation with PPMDQ (vaginal delivery preference) is inverse (p < 0.05, r = -0.324). In other words, increased dissatisfaction with body image is in favor of preferring the method of cesarean delivery.

There was a significant negative correlation between the prenatal body image and all areas of the preferred mode of delivery, except misconceptions about the benefits of cesarean section (p < 0.05). This correlation with the dimensions of self-efficacy (r = -0.290) and willingness to accept (r = -0.266) is stronger than other dimensions of the preferred mode of delivery (r < 0.2) (p < 0.001). Therefore, less dissatisfaction with body image was associated with self-efficacy and more willingness to accept vaginal delivery.

There was a significant negative correlation between preferred mode of delivery and all domains of prenatal body image (p < 0.05). This correlation with the dimensions of beauty and fitness (r = -0.316) and sexual attractiveness (r = -0.312) was stronger than other dimensions (r < 0.3) (p < 0.001). Thus, greater dissatisfaction with fitness and sexual attractiveness was associated with a lower likelihood of choosing vaginal delivery.

**Table 3:** Correlations between Prenatal body image (PBIQ) and its domains and the preferences mode of delivery (PPMDQ) in pregnant women

PPMDQ/PBIQ		Self-efficacy		False impression of the C-section delivery benefits	Exaggerating the risks of vaginal delivery	Perceived susceptibility	Normative believe	Desire for acceptance	Health professionals idea		The total score of the preferences for mode of delivery	
**Fitness and beauty**	r		-0.280	-0.077	-0.168	-0.161	-0.245	-0.208		-0.103	**-0.316**	
	P-value		<0.001	0.173	0.003	0.004	<0.001	<0.001		0.070	**<0.001**	
**Lower body fat**	r		-0.124	-0.064	-0.074	-0.166	-0.082	-0.129		-0.095	**-0.171**	
	P-value		0.029	0.259	0.192	0.003	0.145	0.023		0.093	**-0.002**	
**Attention to changes in pregnancy**	r		-0.167	-0.142	-0.126	-0.122	-0.126	-0.243		-0.161	**-0.257**	
	P-value		0.003	0.012	0.026	0.031	0.026	<0.001		0.004	**<0.001**	
**Shame**	r		-0.198	-0.056	-0.091	-0.041	-0.152	-0.139		-0.096	**-0.203**	
	P-value		<0.001	0.322	0.109	0.473	0.007	0.014		0.089	**<0.001**	
**Sexual attractiveness**	r		-0.280	-0.102	-0.158	-0.134	-0.160	-0.215		-0.099	**-0.312**	
	P-value		<0.001	0.072	0.005	0.017	0.005	<0.001		0.080	**<0.001**	
**Negative feelings about skin changes**	r		-0.148	-0.084	-0.087	-0.171	-0.055	-0.225		-0.093	**-0.203**	
	P-value		0.009	0.140	0.124	0.132	0.329	<0.001		0.101	**<0.001**	
**Symbol of motherhood**	r		-0.285	-0.028	-0.028	-0.023	-0.056	-0.160		-0.107	**-0.190**	
	P-value		<0.001	0.623	0.619	0.689	0.322	0.005		0.058	**<0.001**	
**Prenatal body image**	r		-0.290	-0.102	-0.137	-0.132	-0.150	-0.266		-0.155	**-0.324**	
	P-value		<0.001	0.071	0.015	0.020	0.008	<0.001		0.006	**<0.001**	

**Spearman test**

**r: correlation coefficient**

**P-value: Significance level**

The correlation between the domains of female genital self-image and the domains of the preferred mode of delivery was assessed using Spearman test the results of which are shown in Table 4. There was a weak and significant correlation between the female genital self-image and the preferred mode of delivery (r = 0.198 p = 0.007); so that the women who were more satisfied with their genital image, would choose vaginal mode of delivery. The preferred mode of delivery was significantly correlated with both interpersonal (r = 0.154) and intrapersonal concerns about female genital self-image (r = 0.236) (P < 0.001). Accordingly, increased intrapersonal and interpersonal satisfaction with genital image is associated with the choice of vaginal delivery as the preferred mode of delivery.

**Table 4:** Determining the correlation between the domains of the Female Genital Self-Image (FGSIS) and the domains of the preferences for mode of delivery (PPMDQ) in pregnant women

**PPMDQ**/**FGSIS**		**Self-efficacy**	**False impression of the C-section delivery benefits**	**Exaggerating the risks of vaginal delivery**	**Perceived susceptibility**	**Normative believe**	**Desire for acceptance**	**Health professionals** **idea**	**The total score of the preferences for mode of delivery**
**Interpersonal concerns**	r	0.314	0.095	0.126	0.049	0.035	0.092	-0.008	0.154
	P-value	<0.001	0.094	0.027	0.386	0.540	0.106	0.890	<0.001
**Intrapersonal concerns**	r	0.268	-0.026	0.061	-0.020	0.027	0.090	0.027	0.236
	P-value	<0.001	0.646	0.281	0.724	0.634	0.113	0.637	<0.001
**Genital Self-Image**	r	0.306	0.024	0.108	0.013	0.023	0.095	0.014	0.198
	P-value	<0.001	0.674	0.058	0.818	0.688	0.096	0.801	0.007

Spearman test

r: correlation coefficient

P-value: Significance level

The correlation between the domains of female genital self-image and the domains of prenatal body image was examined using Spearman test the results of which are shown in Table 5. The score of female genital self-image was inversely and significantly correlated with all domains of prenatal body image (P < 0.001).

**Table 5:** Determining the correlation between the domains of Genital Self-Image (FGSIS) and Prenatal body image (PBIQ) in pregnant women

PBIQ/FGSIS		Fitness and beauty	Lower body fat	Attention to changes in pregnancy	Shame	Sexual attractiveness	Negative feelings about skin changes	Symbol of motherhood	Prenatal body image
**Interpersonal concerns**	r	-0.281	-0.170	-0.251	-0.258	-0.309	-0.266	-0.159	-0.332
	P-value	<0.001	0.003	<0.001	<0.001	<0.001	<0.001	0.005	<0.001
**Intrapersonal concerns**	r	-0.238	-0.096	-0.216	-0.224	-0.256	-0.222	-0.128	-0.271
	P-value	<0.001	0.092	<0.001	<0.001	<0.001	<0.001	0.024	<0.001
**Genital Self-Image**	r	-0.279	-0.139	-0.245	-0.272	-0.314	-0.266	-0.152	-0.327
	P-value	<0.001	<0.001	<0.001	<0.001	<0.001	<0.001	<0.001	<0.001

Spearman test

r: correlation coefficient

P-value: Significance level

### Multiple linear regression analysis of FOC

Linear regression model was used to answer this question that “Do body image in pregnancy and female genital self-image predict the preferred mode of delivery?“ The results of Stepwise regression model showed while FGSIS score could not predict PPMDQ, PBIQ score could (beta=-0.173, F = 34.18, P < 0.001). It means that 10 units of increase in PBIQ score can decrease PPMDQ score by 1.7 units. This model explains just 9.7% of response variance (Adjusted R2 = 0.097) (Table 6).

**Table 6:** Linear regression analysis predicting preferred mode of delivery among pregnant women

Variables	B(SE) Unstandardized Coefficients	β (Standardized Coefficients)	P-value
**PBIQ**	-0.173(0.030)	-0.316	< 0.001
**Adjusted R2** **F statistic**	0.097 34.187		

## Discussion

The present study investigated the relationship of the genital image and body image with the preferred mode of delivery. The result showed prenatal body image and female genital image are associated with the choice of vaginal delivery as the preferred mode of delivery.

In the present study the mean of PBIQ was less than average. Most of the participants (89.5%) had a score in the first (completely satisfied) or second quartile (satisfied) based on questionnaire achievable score and no one had a score in the fourth quartile (completely dissatisfied). Exact comparison of the result of this study with others is difficult. It is somehow because different studies using different, non-specific and non-multidimensional measure of body image dissatisfaction during pregnancy [40]. In line with the results of the present study, some studies suggest that, despite gaining weight during pregnancy and staying away from social ideals of beauty, women are satisfied with their body image especially in late pregnancy [41, 25], and pregnancy encourages women to admire and accept the functionality of their body and disregard for self-objectification. Pregnancy is a special time in women’s life when weight gain [25] and appearance changes such as breast and abdomen enlargement are acceptable [3]. Similar to Australian women in Watson et al. [3] study a reason that participants in the present study were mostly satisfied with their body might be they were in third trimester; which pregnancy is well developed and body changes such as rounded abdomen means pregnancy is progressing as it should [3].

However, in a study conducted by Sohrabi et al. in Iran, the mean total score of prenatal body image in pregnant women (in all three trimesters) was higher than the mean score of the present study’s women, meaning that they have been more dissatisfied [29]. Different sampling times can be the reason for this difference in the results of the two studies. The study of Sohrabi et al. [29] was conducted under normal living conditions, but sampling in the present study was performed during the COVID-19 pandemic, when people were encouraged to stay home and restrict their communications [42]. Therefore, during the pandemic, the pregnant women of our study probably experienced less negative emotions caused by changes in body shape and size such as fitness, attention, and shame as they had less interaction with others. In Summary, sampling in late pregnancy and the Covid-19 quarantine period are two main reasons of the high percentage of participants in this study were satisfied about their body. It seems the body changes related to motherhood are perceived similarly in different Western and Eastern cultures [3, 25].

This study demonstrated that most pregnant women were highly satisfied with their genitalia image. Similarly, in a study by Keramat et al. women in their second half of pregnancy, had a FGSIS-7 score higher than the median of the scale range (mean ± SD: 19.98 ± 3.97), although they did not report the FGSIS score distribution based on quartile as we report in this study [39]. However, a comparison of the results with studies conducted on the postpartum or non-pregnant women shows the mean score of the female genital image has been higher in these groups [13, 14, 32, 43]. This difference may be due to the elimination of changes caused by pregnancy and childbirth, and the improvement of women’s body image in the postpartum period. As higher FGSIS is associated with better antenatal mental health [39] and higher overall body image and sexual functioning among women [32], considering this dimension of health in prenatal care could be significant. The FGSIS normal range in pregnant women may differ from non-pregnant as a regular psychological adapt, which warrants caution in interpretation during pregnancy and other life courses.

Based on the quartile distribution of scores of the preferred mode of delivery (PPMD), 2.9% women in the present study definitely preferred cesarean section. This finding is accordance with a meta-analysis showed 3.81% (95% CI 3.74–3.83%) of Iranian women in late pregnancy preferred CS, although the rate was significantly different based on parity, 5.46% of nulliparous women vs. 53.05% for multiparous. Same as the present study which samples were both nullipara and multipara whether the participants were not specified, the proportion of preference for CS was 2.06% [24]. Shargi et al. in Ardabil (a city in northwest of Iran) [44] and Yilmaz et al. in Turkey [45] indicated that the elective mode of delivery was vaginal in 70.7% and 81.5% of women respectively. These results indicate an obvious gap between low preference for CS and high prevalence of cesarean section in Iran that is 2.06% [24] and 47.9% [46] respectively. Multiple individual, health professional, and health system factors affect the preference for CS in Iran. Across studies, the most common individual reasons underlying the preference for CS were tocophobia, fear of pelvic and vaginal damages, and the perceived risks of vaginal delivery for the baby. Financial drives of CS for doctors and the shortage of health systems to provide high-quality care for women who choose vaginal childbirth are among the health system factors that increase the trend in CS rates [24].

In the present study, there was an inverse and significant correlation between the score of prenatal body image and the score of preferred mode of delivery. That is, less dissatisfaction with body image during pregnancy was associated with the choice of vaginal delivery. Similarly, Saadi et al. [20] and Ghahfarokhi et al. [18] showed that weight and appearance concerns are associated with more request for cesarean section.

There was a positive and significant correlation between the score of genital image and the score of preferred mode of delivery in the present study. In other words, increased satisfaction with genital image is related to the preference for vaginal delivery. As shown in the study of Jafarnejad et al., women with vaginal delivery had in their postpartum period a higher score of genital image than women with cesarean section [43]. This result is not in line with the results obtained by Jawed-Wessel et al. They indicated that in their postpartum period, women with a history of vaginal delivery had lower satisfaction with their genital image than women who had a cesarean section. However, it is not clear whether this difference has been related to the type of delivery or has already existed during pregnancy [14].

In the present study, there was a significant inverse correlation between satisfaction with genital image and dissatisfaction with body image in pregnant women, which is consistent with the results of other studies with regard to positive correlation between body image and genital image [11, 13, 14, 19]. However, these studies have not been performed during pregnancy. Nonetheless, bodily changes including breast and abdomen size, and changes in thighs, skin, hair, and weight seem to be more noticeable in pregnant women than genital changes [3].Although some women, because of pregnancy-caused physical changes, do not feel comfortable and confident during and after pregnancy, others find it pleasant and desirable [32]. Similarly, for some women, cesarean section and its complications and in others vaginal delivery, especially if it is accompanied by labor violence, is considered to be a negative experience. Nevertheless, if this experience is accompanied by the creation of a new attitude and a greater sense of empowerment, it will be considered a successful transition which will affect various aspects of their mental health such as body image, genital image and the type of delivery they choose [47].

## Strengths and limitations

One of the strengths of the present study was the use of a special prenatal body image questionnaire, which measures specific characteristics of women’s body image during pregnancy. Given the prevalence of Covid-19 pandemic and decreased visits of pregnant women to comprehensive health centers, the web-based electronic version of the questionnaires was used instead of the paper form for data collection. Among the limitations of electronic and web-based data collection was the lack of participation of women who had not the necessary skills to use smartphones.

## Conclusions and implications for practice

Based on the results of the present study, in pregnant women with a gestational age of 30–37 weeks, dissatisfaction with body image was low and moderate, satisfaction with genital image was high and about half of them certainly or probably preferred cesarean section as a mode of delivery. Moreover, satisfaction with body image and genital image was significantly related with the preferred mode of vaginal delivery. Therefore, improved satisfaction with body image and genital image is expected to be associated with a greater likelihood of choosing vaginal delivery by women.

### Limitations and recommendations

The cut-off point was not specified for the prenatal body image, genital image and preferred mode of delivery method questionnaires. Therefore, in the present study, for better interpretation of the findings, the total score of questionnaires were divided into quartiles. It is suggested that in other studies, the cut-off point of the above questionnaires be determined.

## Electronic supplementary material

Below is the link to the electronic supplementary material.

**Additional file 1**: Appendix table

## Data Availability

The datasets used and/or analyzed during the current study are available from the corresponding author on reasonable request.

## Abbreviations

PBIQ: Prenatal Body Image Questionnaire
FGSIS: Female Genital Self-Image Scale
PPMDQ: Pregnant women’s preferences for mode of delivery questionnaire
DASS-21: Depression, Anxiety and Stress Scale – 21
STROBE: Strengthening the Reporting of Observational studies.
