# Supplementary material for: Preferred mode of delivery association with the body image and genital image in pregnant women - a cross-sectional study
Source: BMC Pregnancy Childbirth. 2023 Jul 4;23:490. doi: 10.1186/s12884-023-05589-3 (PMC10318656; doi:10.1186/s12884-023-05589-3)
Supplement: Supplementary file 1 — Additional file 1: Appendix table [file 12884_2023_5589_MOESM1_ESM.doc]

**Appendix I: The process of sample size estimation (1-3)**

**(1) Sample size estimation:**


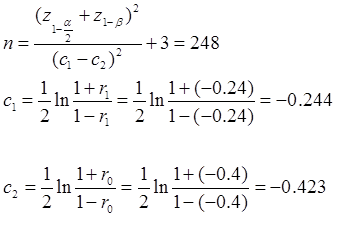


**(2) Sample size estimation based on considering drop out:**

$$n^{*}=\frac{n}{1-f}=\frac{248}{1-0.1}\cong276$$

**(3) Sample size estimation based on considering exclusion based on depression prevalence in pregnancy:**

$$n^{*}=\frac{n}{1-f}=\frac{276}{1-0.15}\cong325$$
